# Getting to the core of cadherin complex function in *Caenorhabditis elegans*

**DOI:** 10.12688/f1000research.6866.1

**Published:** 2015-12-18

**Authors:** Jeff Hardin

**Affiliations:** 1 Department of Zoology, University of Wisconsin-Madison, Madison, WI, USA

**Keywords:** Caenorhabditis elegans, C. elegans, classic cadherin-catenin complex, cadherin, β-catenin

## Abstract

The classic cadherin-catenin complex (CCC) mediates cell-cell adhesion in metazoans. Although substantial insights have been gained by studying the CCC in vertebrate tissue culture, analyzing requirements for and regulation of the CCC in vertebrates remains challenging. *Caenorhabditis elegans* is a powerful system for connecting the molecular details of CCC function with functional requirements in a living organism. Recent data, using an “angstroms to embryos” approach, have elucidated functions for key residues, conserved across all metazoans, that mediate cadherin/β-catenin binding. Other recent work reveals a novel, potentially ancestral, role for the *C. elegans* p120ctn homologue in regulating polarization of blastomeres in the early embryo via Cdc42 and the partitioning-defective (PAR)/atypical protein kinase C (aPKC) complex. Finally, recent work suggests that the CCC is trafficked to the cell surface via the clathrin adaptor protein complex 1 (AP-1) in surprising ways. These studies continue to underscore the value of *C. elegans* as a model system for identifying conserved molecular mechanisms involving the CCC.

## *Caenorhabditis elegans*: a simple system for studying cadherins

The classic cadherin-catenin complex (CCC) is a conserved multiprotein complex found in all metazoans and connects transmembrane cadherins to the actin cytoskeleton ^1^. This strong yet dynamic connection is made possible by linker proteins known as catenins. β-catenin binds the cytoplasmic tail of classic cadherins and binds α-catenin, which in turn binds F-actin. In epithelial tissues *in situ*, the CCC localizes to adherens junctions, which confer adhesive connections between cells and which allow cells to exert forces across epithelia ^2^. In addition, cell-cell contacts must withstand the stresses generated by actomyosin-mediated contractility ^3^. The importance of fundamental work in vertebrate tissue culture for understanding the CCC is well documented and has provided many insights into CCC function. In living organisms, however, the CCC must function in three dimensions and must be tightly regulated. Perhaps the most dramatic example of the need for such regulation is in developing embryos, where cells must make and break cell-cell contacts in a tightly coordinated fashion to build new tissues. Analyzing CCC function in these more complicated tissue environments is challenging; it requires an ability to manipulate key aspects of CCC function while analyzing the detailed cellular effects of such manipulations *in vivo*.

**Figure 1.**
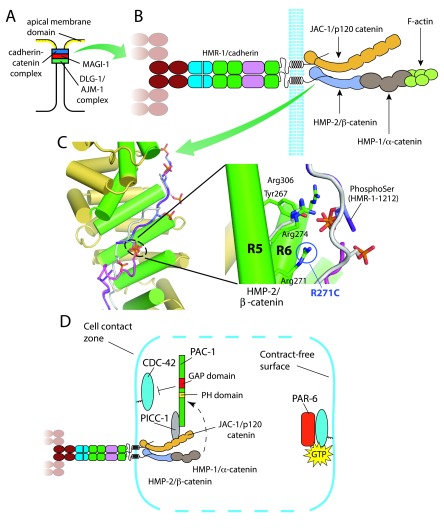
The classic cadherin-catenin complex (CCC) in *Caenorhabditis elegans* is a key regulator of morphogenesis and cell polarity. ( **A**) Schematic of the *C. elegans* apical junction. MAGI-1 (membrane-associated guanylate kinase with inverted organization protein 1) localizes to a domain between the CCC and the DLG-1/AJM-1 complex. ( **B**) The core components of the CCC. HMR-1A is the epithelial cadherin, HMP-2 the junctional β-catenin, HMP-1 the *C. elegans* α-catenin, and JAC-1 the p120ctn homolog. ( **C**) Structures of HMP-2 (Armadillo repeats shown in gold and green) bound to the phosphorylated HMR-1 cytoplasmic domain (magenta). The structure of phosphorylated E-cadherin cytoplasmic domain bound to β-catenin is also shown superimposed on the structure (gray; PDB ID: 1I7W). Phosphoserine 1212 in HMR-1 interacts with residues in HMP-2, including R271, which is mutated to C in the canonical *hmp-2* allele, *zu364*. ( **D**) A model for cadherin-induced cell polarization in blastomeres in the *C. elegans* embryo, based on 41. PAC-1 is recruited via CCC components to cell contact sites, where its GTPase-activating protein (GAP) domain negatively regulates CDC-42 at cell contacts to inhibit recruitment of PAR-6. The dashed line indicates a role that may not be direct. An inferred function that acts independently of the CCC via the pleckstrin-homology (PH) domain of PAC-1 is not shown for clarity. **A** is adapted from 13 with permission. **B** is adapted from 63 with permission. **C** is adapted with permission from a figure courtesy of Hee-Jung Choi by using data from 25.

The nematode *C. elegans* is a powerful tool for *in vivo* analysis of CCC function ^4^. *C. elegans* epithelia possess a *bona fide* adherens junctional complex ( 4, 5; Figure 1A,B), which possesses a single classic cadherin, HMR-1; the short isoform, HMR-1A, forms a complex with HMP-1/α-catenin and HMP-2/β-catenin ^6^ and is bound by the p120ctn homologue, JAC-1, at its juxtamembrane region ^7^. Two key events, driven by morphogenetic movements in the epidermis, critically depend on the CCC. Zygotic loss of function of the cadherin, HMR-1, leads to the Hammerhead phenotype, in which epidermal cells fail to enclose the embryo. Zygotic loss of HMP-1/α-catenin or HMP-2/β-catenin does not lead to enclosure failure, because maternally provided mRNA allows homozygous embryos to complete enclosure. Instead, as the embryo tries to elongate via a coordinated change in shape of its cells from a cuboidal shape to being elongated roughly fourfold along the anterior-posterior axis (reviewed in 8), F-actin-based circumferential filament bundles (CFBs) detach from junctions, leading to the Humpback phenotype. CFB detachment is accompanied by loss or tearing (or both) of the junctional proximal actin network recruited by HMP-1 ^6^.

Remarkably, other earlier embryonic events, such as cleavage and gastrulation, while modulated by CCC function, can proceed without them. The relatively mild resulting phenotypes have actually provided advantages for identifying molecular pathways that act alongside the CCC during cleavage and gastrulation ^9, 10^. During later morphogenesis, the use of weak *hmp-1* and *hmp-2* mutants has similarly made genome-wide RNA interference (RNAi) screens possible to identify molecular components that act with or alongside the CCC during morphogenesis ^11– 14^. This brief review discusses recent work that sheds additional light on the core components of the CCC in *C. elegans*, how the CCC is deployed in the early embryo, and how it is localized during epithelial tissue remodeling.

## A conserved phosphorylation switch controls the cadherin/β-catenin interaction

*C. elegans* is a useful model system for pursuing a structure-function approach to study the CCC. By rescuing strong loss-of-function mutants with green fluorescent protein (GFP)-tagged transgenes, it has been possible to assess several key aspects of CCC function in a living organism. For example, the N-terminal domain of HMP-1/α-catenin, which binds HMP-2/β-catenin, has been shown to be crucial for recruitment of HMP-1 to junctions, and the C terminus, which binds F-actin, is necessary for all key functions of HMP-1 ^15^, vetting results from prior work in tissue culture (reviewed in 16) and consistent with similar recent work in *Drosophila* ^17^.

Because of the mechanical stress experienced by the *C. elegans* epidermis during elongation, its morphogenesis requires robust cell-cell adhesion. However, adhesion in embryos must also be dynamically regulated as cell-cell contacts are extensively remodeled during these processes. One possible mechanism for regulation of CCC components is phosphorylation. Studies in cell culture have revealed that a serine-rich region of the cadherin tail is phosphorylated ^18^, and *in vitro* phosphorylation of the cadherin tail strengthens its affinity for β-catenin by about 800 fold ^19– 21^. Mutation of the phosphorylated serines in the cadherin tail reduces cell-cell adhesion when these constructs are introduced into NIH 3T3 cells ^20^. McEwen *et al*. recently narrowed these phosphorylation sites to three residues that are required for high-affinity β-catenin binding and cell adhesion in cultured cells ^22^. In contrast, phosphorylation of β-catenin by Src at Tyr654 reduces affinity for cadherin ^23^, and CKII phosphorylation of β-catenin regulates its interaction with α-catenin ^24^.

Recent work by Choi *et al*. ^25^ rigorously tests the importance of phosphorylation of the cadherin cytoplasmic tail and β-catenin in *C. elegans*. As with vertebrate E-cadherin, phosphorylation of the HMR-1/cadherin tail dramatically increases its affinity for HMP-2/β-catenin. Solving the structure of an HMR-1/HMP-2 co-crystal reveals that a key conserved serine, S1212, which appears in the structure only when it is phosphorylated, is positioned precisely to allow interaction with several key residues in HMP-2 ( Figure 1C). In some conformations, three additional downstream residues (T1215, S1218, and S1221) are phosphorylated. HMR-1 S1212 and S1218 are equivalent to two of the three residues which have been recently identified as bearing the majority of vertebrate E-cadherin phosphorylation and which have been proposed to be sequentially phosphorylated in tissue culture ^22^. Significantly, S1212 and downstream residues are phosphorylated *in vivo* as well, based on results of co-immunoprecipitation/mass spectrometry analysis performed by Callaci *et al*. ^26^. Independent assessment of binding affinities via reconstitution on liposomes confirms the work of Choi *et al*. showing that S1212 is crucial for HMR-1/HMP-2 association ^26^.

The ideal test of necessity, of course, is *in vivo* requirement. Choi *et al*. found that a full-length *hmr-1::gfp* transgene driven by the endogenous promoter rescues *hmr-1* deletion mutants to viability but that a non-phosphorylatable HMR-1::GFP S1212A construct cannot, despite localizing to junctions ^25^. Although it is difficult to show that sequential phosphorylation actually occurs *in vivo*, Choi *et al*. assessed the possibility that pS1212 acts as a priming site for downstream phosphorylations by creating an *hmr-1(T1215A, S1218A)::gfp* construct in which S1212 remains phosphorylatable, but the C-terminal phosphorylation cascade is blocked. This construct is able to partially rescue *hmr-1(zu389)* embryonic lethality but cannot rescue larval lethality, indicating that, although S1212 is a key phosphoswitch, other phosphosites also have some importance.

In addition to examining the cadherin cytoplasmic tail, Choi *et al*. examined key residues in HMP-2/β-catenin. A gratifying result of this analysis was that sequencing of a canonical strong *hmp-2* allele ( *zu364*) revealed that one of the coordinating residues in HMP-2 visible in the HMR-1/HMP-2 co-crystal, Arg271, is mutated to a cysteine ( Figure 1C). Since previous *in vitro* work had shown that Src phosphorylation of vertebrate β-catenin at Tyr654 reduces its affinity for E-cadherin ^23^, Choi *et al*. ^25^ mutated the equivalent residue in HMP-2, Tyr599, to create phosphomimetic and phospho-null mutations. They found that both constructs were able to rescue *hmp-2(zu364)* to viability and almost as efficiently as wild-type constructs, although the distribution of the phosphomimetic (predicted to have weaker binding to cadherin) becomes punctate and appears to cluster near sites where CFBs insert orthogonally into the junctional-proximal actin network. This suggests that the CCC in such embryos may be more susceptible to mechanical tension during morphogenesis, since CFBs are thought to transmit tension throughout the epidermis ^8^.

In addition to the sites identified in the work of Choi *et al*., Callaci *et al*. found phosphosites in HMP-2 and HMP-1 in embryonic extracts. *In vitro*, HMP-2 protein harboring phosphomimetic mutations at the sites identified from extracts reduced its affinity for HMR-1. *In vivo*, constructs carrying identical phosphomimetic mutations rescue, but somewhat more weakly than wild-type transgenes. In contrast, perturbations of phosphosites identified in HMP-1 from embryonic extracts did not affect the ability of HMP-1 to bind actin or its comformation ^26^. These results are similar to those in vertebrate tissue culture and *Drosophila*, which found only weak effects of numerous phosphosites in α-catenin ^27^.

Taken together, these results demonstrate the fruitfulness of an “angstroms to embryos” approach in *C. elegans* (i.e., correlation of changes in single residues identified as important from detailed molecular structural studies with aggregate effects on embryogenesis). There are several limitations to this approach, however. First, it leaves an important “meso-level” analysis of CCC function, such as the role of subcellular mechanics, largely unexplored (e.g., 28). Second, basic structure-function studies cannot address dynamic phosphorylation and dephosphorylation events. It remains to be seen whether such dynamic events can fine-tune CCC function or whether the functionally important sites identified in these studies are constitutively phosphorylated.

## The PAR/aPKC complex as an upstream regulator of the CCC

There is abundant evidence that cadherin localization in metazoan epithelia is heavily influenced by molecular pathways that establish and maintain apicobasal polarity. In particular, polarity crucially depends on the function of the PAR/aPKC complex ^29– 31^. PAR proteins PAR-3 and PAR-6 were originally discovered in *C. elegans* through their maternal role in partitioning components in the one-cell zygote ^32^. Subsequent work in worms, *Drosophila*, and tissue culture added molecular players and further clarified the role of this important complex in the establishment of cell polarity. A conserved cassette includes activated Cdc42, which binds Par6, which in turn recruits aPKC. Par3 (Bazooka in *Drosophila*) and Cdc42/Par6/aPKC engage in a complicated interplay at the apical ends of cells in many contexts (reviewed in 29, 31, 33– 35).

Recent work in *C. elegans* has added insight into PAR/aPKC function, especially as it relates to CCC function. Previous work had shown that during *C. elegans* intestinal differentiation, CCC and polarity complex proteins colocalize to foci that accumulate at the apical surface ^36, 37^, eventually coalescing to form *bona fide* junctions. Examining the role of the PAR/aPKC complex during this process was made possible by using maternally provided, modified PAR-3 and PAR-6 proteins that are present during polarization of the one-cell zygote but that are rapidly degraded thereafter ^37^. Curiously, in the absence of PAR-3, intestinal HMR-1 is initially mislocalized, but HMP-1 is still present in foci ^36^ that now localize with the more basal junctional component, DLG-1/Discs large ^38^. Similar localization defects are seen in the pharynx in *par-3* mutants ^36^. Although *par-3* function is initially dispensable in the epidermis, apical localization of HMR-1 and DLG-1 is progressively lost and the epidermis eventually tears ^36^. In both the intestine and the epidermis, PAR-6 is required for junctional maturation but not for targeting of the CCC to initial spot junctions ^37^. This result is largely consonant with similar previous work in *Drosophila* showing that the relationship between Par3 and Par6 is complex. There, Par3/Bazooka is initially upstream of Par6, but later Par3 and Par6/aPKC become spatially separated (reviewed in 35).

Recent work on the *C. elegans* pharynx extends previous work by showing that PAR-6 is necessary for polarization of arcade cells ^39^. HMR-1/cadherin, the ERM protein, ERM-1, and the DLG-1/Discs large binding partner, AJM-1, all fail to localize in *par-6*(M/Z) embryos. In contrast to the crucial requirement for *par-6* function, embryos knocked down for *hmr-1*/cadherin and *pat-3*/β-integrin do not show major defects in localization of apical proteins, suggesting that neither the CCC nor adhesion to the basal lamina is essential for apical polarization in arcade cells. In the future, identifying other cell adhesion pathways that act in the absence of these two crucial adhesion systems should yield insights into how epithelia regulate polarity downstream of the PAR/aPKC system in metazoa.

## The CCC as a reciprocal regulator of the PAR/aPKC complex in blastomeres

The relationship between the CCC and the PAR/aPKC proteins, based on the work described thus far, is admittedly somewhat complex in detail but comports well with work in many other systems, in which the PAR/aPKC complex acts upstream of the CCC in epithelial polarization (reviewed in 29, 31, 33). It is surprising, then, that the PAR/aPKC complex is not essential for blastomere adhesion in the early embryo ^38^. This lack of absolute requirement, however, has proven to be an advantage for teasing apart the complex interplay between the CCC and the PAR/aPKC complex during embryogenesis. To gain insight into how blastomere polarity is established, Anderson *et al.* ^40^ screened for mutations that prevent the normal localization pattern of PAR-6::GFP, which is confined to the outer (non-contact) surfaces of blastomeres in early *C. elegans* embryos. They identified PAC-1 (ARHGAP21 in humans), a Rho-family GTPase-activating protein (GAP). PAR-6, PAR-3, and aPKC/PKC-3 all show a symmetric cortical localization in *pac-1* blastomeres. As in embryos with M/Z loss of PAR proteins, *pac-1* mutant embryos show delayed ingression of cells during gastrulation but no defects in epithelial polarity in the later embryo, suggesting that PAC-1 is dispensable for other types of PAR asymmetries. GFP::PAC-1 localizes to inner (contact) surfaces but not the outer surfaces of blastomeres (i.e., in a pattern complementary to PAR-6). The complementary distributions of PAR-6 and PAC-1 suggest that PAC-1 controls PAR-6 localization by inhibiting CDC-42 at contact surfaces between blastomeres. This supposition was confirmed by making mutant PAR-6 protein that cannot bind CDC-42. The mutant protein mislocalizes, indicating that activated CDC-42 leads to recruitment of PAR-6, whereas PAC-1 at cell-cell contacts prevents PAR-6 accumulation there through downregulation of CDC-42 ^40^.

Recent work from the Nance group extends this PAC-1/ARHGAP21 story ^41^. Given that PAC-1 localizes to the same sites in the early embryo where the CCC is found, Klompstra *et al*. ^41^ examined the distribution of full-length PAC-1 in *hmr-1*/cadherin loss-of-function embryos and found that it is reduced, although its localization is not abolished. N-terminal fragments of PAC-1 completely fail to localize in *hmr-1* mutants, indicating that this region of the protein is regulated by the CCC. Deleting the pleckstrin-homology (PH) domain of PAC-1 abolished this residual PAC-1 in *hmr-1* mutant embryos, indicating that this domain is required for the putative redundant localization mechanism. Taken together, these results suggest that both the CCC and other unidentified mechanisms localize PAC-1 to contact sites between blastomeres.

Klompstra *et al*. then examined the catenins. Loss of *jac-1*/p120ctn function led to partial loss of GFP–PAC-1N from cell contacts. Removing HMP-1 in *jac-1* embryos caused a complete loss of GFP–PAC-1N from contacts, as in *hmr-1* embryos; these results suggest that JAC-1 and HMP-1 may work together in localizing PAC-1. Yeast two-hybrid screening eventually identified a possible mechanism linking PAC-1 and JAC-1 involving PICC-1 (PAC-1-interacting coiled-coil protein 1), the homologue of a poorly characterized human coiled-coil protein known as CCDC85B/DIPA, which has been shown to bind p120ctn ^42^. GFP::PICC-1 localizes in an HMR-1- and JAC-1-dependent manner but persists in *hmp-1(RNAi)* embryos. Taken together, these data suggest a model in which PAC-1 is localized to cell surfaces in contact via both JAC-1/PICC-1 and HMP-1 ( Figure 1D). To test this CCC-dependent model for Cdc42/PAR polarization, Klompstra *et al*. forced expression of the HMR-1 cytoplasmic tail ubiquitously at the cell surface by using a PH-domain chimera. The result was loss of polarization and greatly reduced PAR-6 at the cortex. These studies suggest a model whereby CCC-dependent contact between blastomeres leads to localized downregulation of Cdc42 via PAC-1/ARHGAP21 ( Figure 1D).

The findings of Klompstra *et al*. may also help to explain puzzling results from the previous literature. As stated above, phenotypes associated with loss of function of the single p120ctn family member in both flies and worms are remarkably mild, given the clearly essential roles that p120ctn plays in vertebrates ^43– 46^. One potential explanation for this discrepancy is that the δ-catenin/p120ctn gene family in vertebrates has apparently arisen because of gene duplication from a single ancestral gene that encoded a protein more similar to δ-catenin. Subsequently, a gene encoding p120ctn arose in vertebrates, which has evolved essential roles in numerous processes (reviewed in 47). The work of Klompstra *et al*. may have uncovered an ancestral role for p120ctn/δ-catenin in spatial control of GAPs for Rho family GTPases. These findings comport well with previous studies of p120ctn, which have implicated it in recruitment of p190RhoGAP to junctions ^48– 51^. However, in worms, the role of PAC-1 is modulatory, since loss of *pac-1* function is not lethal on its own ( 40 and Jeff Hardin and Allison Lynch, unpublished data).

## The AP-1 complex regulates localization of the CCC during tissue remodeling

In addition to regulation of the core components of the cadherin/catenin complex by phosphorylation and through interactions with the partitioning-defective (PAR)/atypical protein kinase C (aPKC) polarity system, a well-known regulatory node affecting the CCC is intracellular trafficking. Here again, much of this work has been done in cultured cells (reviewed in 52– 54); *C. elegans* has promise as a system for examining what aspects of trafficking of CCC components to and from the plasma membrane are essential in a metazoan. Some previous results have been surprising in the context of endocytic recycling of cadherins. For example, p120ctn has been shown to regulate endocytic trafficking of VE-cadherin by masking a conserved motif in its cytoplasmic tail ^55^. If such regulation occurs in the case of HMR-1/cadherin in *C. elegans*, it must only be modulatory, given the mild phenotypes observed in the absence of JAC-1/p120ctn ^7, 41^. Similar results have been obtained in *Drosophila*, in which very mild phenotypes have been reported under laboratory conditions following p120ctn loss of function ^56, 57^. The AP-2 clathrin adaptor complex is likewise surprisingly dispensable in *C. elegans* ^58, 59^, although loss of AP-2 components has been reported to lead to synergistic lethality in a weak *hmp-1*/α-catenin mutant background in a genome-wide RNAi screen ^13^.

Work from the Michaux group ^60^ has shown that, in contrast to the AP-2 complex, which is involved in endocytic recycling, the clathrin adaptor protein complex 1 (AP-1), well known to be involved in the export of proteins to the plasma membrane, is required for normal apicobasal polarity in the embryonic intestine of *C. elegans*. Knockdown of AP-1 subunits leads to homogeneous distribution of apical and basally localized proteins, including, significantly, PAR/aPKC complex proteins and CDC-42. Interestingly, knockdown embryos have ectopic lumenal vesicles, including components of both the CCC and the more basal complex that contains the *C. elegans* homologue of Discs large, DLG-1, and its binding partner, AJM-1 ^60^.

Recent work by the Michaux group ^61^ extends this work on AP-1 to the *C. elegans* epidermis. To identify genes required for polarized localization of HMR-1 in the epidermis, Gillard *et al*. ^61^ used RNAi of candidate genes to identify genes whose knockdown led to lateral mislocalization of HMR-1. These included *chc-1*/clathrin heavy chain, the σ and γ subunits of AP-1 complex ( *aps-1* and *apg-1*), the functionally redundant AP-1 μ subunits, and the AP-1-interacting protein HEATR5B/p200/Laa1p (in *C. elegans* known as *soap-1*, for sorting of apical proteins). In contrast, depletion of dynamin, RAB-5, RAB-11, or exocyst complex components led to changes in overall levels of HMR-1 but not its apicobasal localization. Thus, although these proteins play an essential role in HMR-1 secretion or recycling (or both) at the apical membrane, they—unlike AP-1 components—are not involved in apicobasal polarity. Gillard *et al*. went on to examine interactions among AP-1, clathrin subunits, and SOAP-1. Loss of AP-1 led to a strong decrease in recruitment of clathrin subunits to membranes. Similarly, loss of SOAP-1 led to an overall reduction in AP-1 components, suggesting that SOAP-1 is involved in targeting of AP-1 to sites of protein export to the membrane.

Additional observations, however, suggest that there are more global disruptions in apicobasal polarization in epidermal cells following loss of AP-1 components that include, but are not restricted to, the CCC. RNAi against the σ, γ, or both μ subunits of AP-1 induces a shift in the position of the DLG-1/AJM-1 complex to a more lateral position. In addition, in normal embryos, a small amount of HMR-1/cadherin is visible in the lateral membrane, but this lateral cadherin disappears during subsequent morphogenesis as the embryo elongates. In contrast, in *aps-1(RNAi)* embryos, HMR-1 accumulates laterally. Given the crucial role of the CCC during morphogenesis, this leads to disruption of F-actin during elongation. How the AP-1 is distinctively involved in maintenance of apicobasal polarity remains unclear.

Taken together, the findings of Gillard *et al*. also raise interesting questions regarding the selectivity of effects of perturbing the AP-1 complex and other apicobasal cues in various epithelia in *C. elegans*. The specific mislocalization of HMR-1 in the epidermis, for example, contrasts markedly with the effects of depletion of AP-1 component in the intestine, in which no such mislocalization is observed. Previous work on the PAR complex similarly showed differential requirements in different tissues. While PAR-3 is critical for CCC establishment in the intestine, it is surprisingly dispensable in the epidermis, although localization of HMR-1 and DLG-1 is eventually lost, along with the mechanical integrity of the epidermis ( 36; reviewed in 62). What might account for such differences? The answer is unclear, but a key difference between the intestine and epidermis is the presence of a cuticle in the latter. Identifying molecular differences in CCC trafficking and apicobasal polarization in these tissues will be interesting in the future.

## Conclusions

*C. elegans* is a useful system for studying both the core functions and regulation of the CCC. On the one hand, the rapidity with which the function of single amino acids within each of the core CCC proteins can be assessed has allowed incisive experiments to be performed that link single amino acid changes in CCC proteins to essential, highly specific morphogenetic processes. On the other hand, the surprising lack of stringent requirements for CCC components or their modifiers in some developmental events has provided an opportunity to uncover conserved pathways shared by all metazoans. There is every reason to expect *C. elegans* to continue to be a useful model system for analyzing the CCC in the future.

## Abbreviations

AP-1, clathrin adaptor protein complex 1; aPKC, atypical protein kinase C; CCC, cadherin-catenin complex; CFB, circumferential filament bundle; GAP, GTPase-activating protein; GFP, green fluorescent protein; PAR, partitioning-defective; PH, pleckstrin-homology; RNAi, RNA interference; SOAP-1, sorting of apical proteins-1.
